# Dragon 1 Protocol Manuscript: Training, Accreditation, Implementation and Safety Evaluation of Portal and Hepatic Vein Embolization (PVE/HVE) to Accelerate Future Liver Remnant (FLR) Hypertrophy

**DOI:** 10.1007/s00270-022-03176-1

**Published:** 2022-07-05

**Authors:** R. Korenblik, B. Olij, L. A. Aldrighetti, M. Abu Hilal, M. Ahle, B. Arslan, L. J. van Baardewijk, I. Baclija, C. Bent, C. L. Bertrand, B. Björnsson, M. T. de Boer, S. W. de Boer, R. P. H. Bokkers, I. H. M. Borel Rinkes, S. Breitenstein, R. C. G. Bruijnen, P. Bruners, M. W. Büchler, J. C. Camacho, A. Cappelli, U. Carling, B. K. Y. Chan, D. H. Chang, J. choi, J. Codina Font, M. Crawford, D. Croagh, E. Cugat, R. Davis, D. W. De Boo, F. De Cobelli, J. F. De Wispelaere, O. M. van Delden, M. Delle, O. Detry, R. Díaz-Nieto, A. Dili, J. I. Erdmann, O. Fisher, C. Fondevila, Å. Fretland, F. Garcia Borobia, A. Gelabert, L. Gérard, F. Giuliante, P. D. Gobardhan, F. Gómez, T. Grünberger, D. J. Grünhagen, J. Guitart, J. Hagendoorn, J. Heil, D. Heise, E. Herrero, G. F. Hess, M. H. Hoffmann, R. Iezzi, F. Imani, J. Nguyen, E. Jovine, J. C. Kalff, G. Kazemier, T. P. Kingham, J. Kleeff, O. Kollmar, W. K. G. Leclercq, S. Lopez Ben, V. Lucidi, A. MacDonald, D. C. Madoff, S. Manekeller, G. Martel, A. Mehrabi, H. Mehrzad, M. R. Meijerink, K. Menon, P. Metrakos, C. Meyer, A. Moelker, S. Modi, N. Montanari, J. Navines, U. P. Neumann, P. Peddu, J. N. Primrose, X. Qu, D. Raptis, F. Ratti, F. Ridouani, C. Rogan, U. Ronellenfitsch, S. Ryan, C. Sallemi, J. Sampere Moragues, P. Sandström, L. Sarriá, A. Schnitzbauer, M. Serenari, A. Serrablo, M. L. J. Smits, E. Sparrelid, E. Spüntrup, G. A. Stavrou, R. P. Sutcliffe, I. Tancredi, J. C. Tasse, V. Udupa, D. Valenti, Y. Fundora, T. J. Vogl, X. Wang, S. A. White, W. A. Wohlgemuth, D. Yu, I. A. J. Zijlstra, C. A. Binkert, M. H. A. Bemelmans, C. van der Leij, E. Schadde, R. M. van Dam

**Affiliations:** 1 GROW School for Oncology and Developmental Biology, Maastricht University, Maastricht Universiteitssingel 40 room 5.452, 6229 ET Maastricht, The Netherlands; 2 Department of Surgery, Maastricht University Medical Center, Maastricht, The Netherlands; 3 Deparment of Radiology and Nuclear Medicine, Maastricht University Medical Center, Maastricht, The Netherlands; 4 Department of General, Visceral and Transplant Surgery, University Hospital Aachen, Aachen, Germany; 5 Department of Radiology, University Hospital Aachen, Aachen, Germany; 6 Department of General and Visceral Surgery, Cantonal Hospital Winterthur, Winterthur, Switzerland; 7 Department of Radiology, Cantonal Hospital Winterthur, Winterthur, Switzerland; 8 Department of Radiology, Rush University Medical Center, Chicago, USA; 9 Department of Surgery, Rush University Medical Center Chicago, Chicago, USA; 10 Department of Surgery, Ospedale San Raffaele, Milan, Italy; 11 Department of Surgery, Fondazione Poliambulanza, Brescia, Italy; 12 Deparment of Radiology, University Hospital, Linköping, Sweden; 13 Department of Radiology, Maxima Medisch Centrum, Eindhoven, The Netherlands; 14 Department of Radiology, Clinic Favoriten, Vienna, Austria; 15 Department of Radiology, Bournemouth and Christuchurch, The Royal Bournemouth and Christchurch Hospitals, Bournemouth and Christuchurch, UK; 16 Department of Surgery, CHU UCLouvain Namur, Namur, Belgium; 17 Department of Surgery, Biomedical and Clinical Sciences, Linköping University Hospital, Linköping, Sweden; 18 Department of Surgery, University Medical Center Groningen, Groningen, The Netherlands; 19 Department of Radiology, University Medical Center Groningen, Groningen, The Netherlands; 20 Department of Surgery, University Medical Center Utrecht, Utrecht, The Netherlands; 21 Department of Radiology, University Medical Center Utrecht, Utrecht, The Netherlands; 22 Department of Surgery, University Hospital Heidelberg, Heidelberg, Germany; 23 Department of Radiology, Memorial Sloan Kettering Cancer Center, New York, USA; 24 Department of Radiology, IRCCS Azienda Ospedaliero-Universitaria di Bologna, Sant’Orsola-Malpighi Hospital, Bologna, Italy; 25 Department of Radiology, University Hospital Oslo, Oslo, Norway; 26 Department of Surgery, Aintree University Hospitals NHS, Liverpool, UK; 27 Department of Radiology, University Hospital Heidelberg, Heidelberg, Germany; 28 Department of Surgery, Western Health Footscray, Footscray, Australia; 29 Department of Radiology, University Hospital Dr. Josep Trueta de Girona, Girona, Spain; 30 Department of Surgery, Royal Prince Alfred Hospital, Camperdown, Australia; 31 Department of Surgery, Monash Health, Clayton, Australia; 32 Department of Surgery, University Hospital Germans Trias I Pujol, Badalona, Spain; 33 Department of Radiology, Aintree University Hospitals NHS, Liverpool, UK; 34 Department of Radiology, Monash Health, Clayton, Australia; 35 Department of Radiology, Ospedale San Raffaele, Milan, Italy; 36 Department of Radiology, CHU UCLouvain Namur, Namur, Belgium; 37 Department of Radiology, Amsterdam University Medical Centers Location AMC, Amsterdam, The Netherlands; 38 Department of Radiology, Karolinska University Hospital, Stockholm, Sweden; 39 Department of Surgery, CHU de Liège, Liège, Belgium; 40 Department of Surgery, Amsterdam University Medical Centers Location AMC, Amsterdam, The Netherlands; 41 Department of Surgery, Hospital Clínic de Barcelona, Barcelona, Spain; 42 Department of Surgery, University Hospital Oslo, Oslo, Norway; 43 Department of Surgery, Hospital Parc Taulí de Sabadell, Sabadell, Spain; 44 Department of Radiology, Hospital Parc Taulí de Sabadell, Sabadell, Spain; 45 Department of Radiology, University Hospital Mútua Terassa, Terassa, Spain; 46 Department of Radiology, CHU de Liège, Liège, Belgium; 47 Department of Surgery, Gemelli University Hospital Rome, Rome, Italy; 48 Department of Surgery, Amphia, Breda, The Netherlands; 49 Department of Radiology, Hospital Clínic de Barcelona, Barcelona, Spain; 50 Department of Surgery, HPB Center Vienna Health Network, Clinic Favoriten, Vienna, Austria; 51 Department of Surgery, Erasmus Medisch Centrum, Rotterdam, The Netherlands; 52 Department of Surgery, University Hospital Frankfurt, Frankfurt, Germany; 53 Department of Surgery, University Hospital Mútua Terassa, Terassa, Spain; 54 Department of Surgery, Clarunis University Hospital, Basel, Switzerland; 55 Department of Radiology, St. Clara Spital, Basel, Switzerland; 56 Department of Radiology, Gemelli University Hospital, Rome, Italy; 57 Department of Radiology, Amphia, Breda, The Netherlands; 58 Department of Radiology, Western Health Footscray, Footscray, Australia; 59 Department of Surgery, Ospedale Maggiore di Bologna, Bologna, Italy; 60 Department of Surgery, University Hospital Bonn, Bonn, Germany; 61 Department of Surgery, Amsterdam University Medical Centers Location VU, Amsterdam, The Netherlands; 62 Department of Surgery, Memorial Sloan Kettering Cancer Center, New York, USA; 63 Department of Surgery, University Hospital Halle (Saale), Halle, Germany; 64 Department of Surgery, Maxima Medisch Centrum, Eindhoven, The Netherlands; 65 Department of Surgery, University Hospital Dr. Josep Trueta de Girona, Girona, Spain; 66 Department of Surgery, Hôpital Erasme, Brussels, Belgium; 67 Department of Radiology, Oxford University Hospital NHS, Oxford, UK; 68 Department of Radiology, Yale School of Medicine, New Haven, USA; 69 Department of Surgery, The Ottawa Hospital, Ottawa, Canada; 70 Department of Radiology, Queen Elizabeth Hospital Birmingham NHS, Birmingham, UK; 71 Department of Radiology, Amsterdam University Medical Centers Location VU, Amsterdam, The Netherlands; 72 Department of Surgery, King’s College Hospital NHS, London, UK; 73 Department of Surgery, McGill University Health Centre, Montréal, Canada; 74 Department of Radiology, University Hospital Bonn, Bonn, Germany; 75 Department of Radiology and Nuclear Medicine, Erasmus Medisch Centrum, Rotterdam, The Netherlands; 76 Department of Radiology, University Hospital Southampton NHS, Southampton, UK; 77 Department of Radiology, Ospedale Maggiore Di Bologna, Bologna, Italy; 78 Department of Radiology, King’s College Hospital NHS, London, UK; 79 Department of Surgery, University Hospital Southampton NHS, Southampton, UK; 80 Department of Radiology, Zhongshan Hospital, Fundan University, Shanghai, China; 81 Department of Surgery, Royal Free Hospital NHS, London, UK; 82 Department of Radiology, Royal Prince Alfred Hospital, Camperdown, Australia; 83 Department of Radiology, The Ottawa Hospital, Ottawa, Canada; 84 Department of Radiology, Fondazione Poliambulanza, Brescia, Italy; 85 Department of Radiology, University Hospital Germans Trias I Pujol, Badalona, Spain; 86 Department of Radiology, University Hospital Miguel Servet, Saragossa, Spain; 87 Department of Surgery, General Surgery and Transplant Unit, IRCCS Azienda Ospedaliero- Universitaria di Bologna, Sant’Orsola-Malpighi Hospital, Bologna, Italy; 88 Department of Surgery, University Hospital Miguel Servet, Saragossa, Spain; 89 Department of Surgery, Karolinska University Hospital, Stockholm, Sweden; 90 Department of Radiology, Klinikum Saarbrücken gGmbH, Saarbrücken, Germany; 91 Department of Surgery, Klinikum Saarbrücken gGmbH, Saarbrücken, Germany; 92 Department of Surgery, Queen Elizabeth Hospital Birmingham NHS, Birmingham, UK; 93 Department of Radiology, Hôpital Erasme, Brussels, Belgium; 94 Department of Surgery, Oxford University Hospital NHS, Oxford, UK; 95 Department of Radiology, McGill University Health Centre, Montréal, Canada; 96 Department of Radiology, University Hosptital Frankfurt, Frankfurt, Germany; 97 Department of Surgery, Zhongshan Hospital, Fundan University, Shanghai, China; 98 Department of Surgery, Newcastle Upon Tyne Hospitals NHS, Newcastle upon Tyne, UK; 99 Department of Radiology, University Hospital Halle (Saale), Halle, Germany; 100 Department of Radiology, Royal Free Hospital NHS, London, UK

**Keywords:** Colorectal cancer liver metastases (CRLM), Portal vein embolization (PVE), Hepatic vein embolization (HVE), Combined portal- and hepatic vein embolization (PVE/HVE), Liver hypertrophy, Future liver remnant (FLR)

## Abstract

**Study Purpose:**

The DRAGON 1 trial aims to assess training, implementation, safety and feasibility of combined portal- and hepatic-vein embolization (PVE/HVE) to accelerate future liver remnant (FLR) hypertrophy in patients with borderline resectable colorectal cancer liver metastases.

**Methods:**

The DRAGON 1 trial is a worldwide multicenter prospective single arm trial. The primary endpoint is a composite of the safety of PVE/HVE, 90-day mortality, and one year accrual monitoring of each participating center. Secondary endpoints include: feasibility of resection, the used PVE and HVE techniques, FLR-hypertrophy, liver function (subset of centers), overall survival, and disease-free survival. All complications after the PVE/HVE procedure are documented. Liver volumes will be measured at week 1 and if applicable at week 3 and 6 after PVE/HVE and follow-up visits will be held at 1, 3, 6, and 12 months after the resection.

**Results:**

Not applicable.

**Conclusion:**

DRAGON 1 is a prospective trial to assess the safety and feasibility of PVE/HVE. Participating study centers will be trained, and procedures standardized using Work Instructions (WI) to prepare for the DRAGON 2 randomized controlled trial. Outcomes should reveal the accrual potential of centers, safety profile of combined PVE/HVE and the effect of FLR-hypertrophy induction by PVE/HVE in patients with CRLM and a small FLR.

**Trial Registration:**

Clinicaltrials.gov: NCT04272931 (February 17, 2020). Toestingonline.nl: NL71535.068.19 (September 20, 2019).

**Supplementary Information:**

The online version contains supplementary material available at 10.1007/s00270-022-03176-1.

## Introduction

### Background and Rationale

Removal of colorectal liver metastases (CRLM) has been shown to improve survival of patients with stage IV colorectal cancer. However, many patients with multifocal liver metastases require resections that might put them at risk of post-hepatectomy liver failure (PHLF) [1]. When resection of more than 70% of functional liver volume in normal functioning livers or more than 60% in damaged livers is necessary, patients are at high risk of developing PHLF, which increases the risk of perioperative mortality [2]. These patients are therefore often considered primarily unresectable or potentially resectable (PU/PR), based on computed tomography volumetry of the future liver remnant (FLR) [3]. The most commonly applied method to avoid PHLF is to induce hypertrophy of the FLR before surgery, usually by portal vein embolization (PVE) [4].

PVE involves the embolization of the portal venous system to one side of the liver, inducing growth of the other side (FLR). After PVE, an FLR increase up to 40% can be observed after 3–6 weeks [5]. However, several studies showed that only 60–70% of patients underwent hepatectomy after PVE [6–10], due to insufficient hypertrophy or disease progression. Interest has consequently focused on the question whether rapid hypertrophy can be induced without a two-stage hepatectomy such as Associating Liver Partition and Portal Vein Ligation for Staged Hepatectomy (ALPPS, supplementary paragraph 1) [11–15].

Right hepatic vein embolization following PVE was first described in a case report in 2002 by Nagino et al. showing the applicability of the technique [16]. Consequently, small cohort studies were performed to investigate this combined procedure [17]. Experiments in pigs showed that an abrogation of hepatic vein outflow from the deportalized side accelerates regeneration similar to ALPPS [18]. All of these findings led to the development of a novel clinical approach to induce liver growth by combined Portal and Hepatic Vein Embolization (PVE/HVE). Guiu et al. performed the first variation adding glue to the PVE/HVE procedure, Liver Venous Deprivation (LVD), in humans [19]. They showed that FLR increased from 28.2% (range 22.4–33.3%) to 40% (33.6–59.3%) 23 days after this procedure with the largest increase in the first 7 days [19, 20].

To assess the clinical value of PVE/HVE in patients eligible for extended liver resection and small future liver remnants, and to safely implement a new technique, the worldwide DRAGON collaborative was initiated in 2017.

## Methods

### Objectives

The primary objective of DRAGON 1 is to assess the safety of the PVE/HVE procedure together with obtaining insight in the accrual ability of each individual center. Structured training in the novel technique should increase safety and allow for initial experience for those centers unfamiliar with the procedure in the DRAGON 1 trial.

Secondary objectives of DRAGON 1 are to assess the efficacy of PVE/HVE and the different PVE/HVE techniques. The latter to optimize the procedure prior to the DRAGON 2 trial.

### Study Setting/Design

The DRAGON 1 trial is an International Multicenter trial for safety and feasibility evaluation of PVE/HVE. Most of the participating centers are Academic Hospitals. For a detailed list of the participating countries and their study sites see supplementary table 1.

In the DRAGON 1 trial, PVE/HVE will only be performed in patients with primarily unresectable/ potentially resectable (PU/PR) Colorectal Cancer Liver Metastases (CRLM). The total study duration for a center in the DRAGON trial 1 will be 24 months, with 12 months inclusion and 12 months follow-up. For each center, the inclusion phase will last a year after the first enrollment. The follow-up phase will last a year after the second stage resection of the last patient. Patients who do not proceed to surgical resection after PVE/HVE will also be routinely assessed until one year after combined PVE/HVE.

### Eligibility

All participating centers must obtain local ethical review board approval, and if needed according to local regulations, radiation protection approval. Centers can apply for enrollment if, based on center volume, the minimum number of inclusions of three patients within one year can be achieved. International participants’ Insurance is provided by the sponsor. Patients diagnosed with PU/PR colorectal liver metastases will be recruited via referral from the oncology, surgery, IR clinics, and local tumor boards of the participating centers. The inclusion and exclusion criteria are displayed in Table 1.

**Table 1:** Inclusion and exclusion criteria

Inclusion criteria	Exclusion criteria
Patients with primarily unresectable/potentially resectable colorectal liver metastases and a small future liver remnant (< 30% in normal livers or < 40% in chemotheraphy demaged livers)	Patients who did not receive conversion chemotherapy
Age > 18 years	Patients with extrahepatic disease who can’t be curatively treated
Eastern Cooperative Oncology Group performance status < 3 (not more than 50% bedbound)	Patients with extrahepatic disease who can’t be curatively treated
Patients with non-resected primary colorectal cancer (CRC) may be included but only when the intention is to remove the CRC after the lover treatment (liver first approach)	Pregnant or lactating women of conceiving age are required to take contraceptives or provide documentation of other means of contraception
No unresectable extrahepatic disease (metastatic disease that can be curatively treated in included)	Progression by modified RECIST criteria on cross-sectional imaging after conversion chemotherapy
Patients must be able to understand the trial and provide informed consent	Complete response in cross-sectional imaging after conversion chemotherapy

### Intervention

In combined PVE/HVE, the portal vein branch of one side of the liver and hepatic vein(s) draining the same side will be occluded to induce hypertrophy on the contralateral side [21]. PVE is performed according to local standard practice, with technical modifications between centers being allowed, to assess optimal approach for the DRAGON 2 trial. The PVE-technique used will be registered. Once access to the target portal vein has been obtained, the vein will be occluded using either a mixture of Lipiodol/cyanoacrylate, particles and coils or other embolization materials, according to local practice. After the procedure, the access sheath is retracted and the track occluded. Subsequently, HVE is performed in the same session or within 48 h using either a trans-jugular approach, a trans-hepatic approach or a transfemoral approach, according to the preference of the interventionist. Through a sheath, appropriately sized Amplatzer Vascular Plug(s) (type I,II, or IV) are introduced into the draining (usually right and sometimes middle) hepatic vein branches of the affected liver side. The number of hepatic veins to be occluded is left to the local team and depends on the individual anatomy of the liver. At least one large draining vein must be occluded.

All procedures were defined in centrally designed Work Instructions (WI) improving adherence to the interventions and subsequent study tasks.

### Participant Timeline

**Fig. 1:**
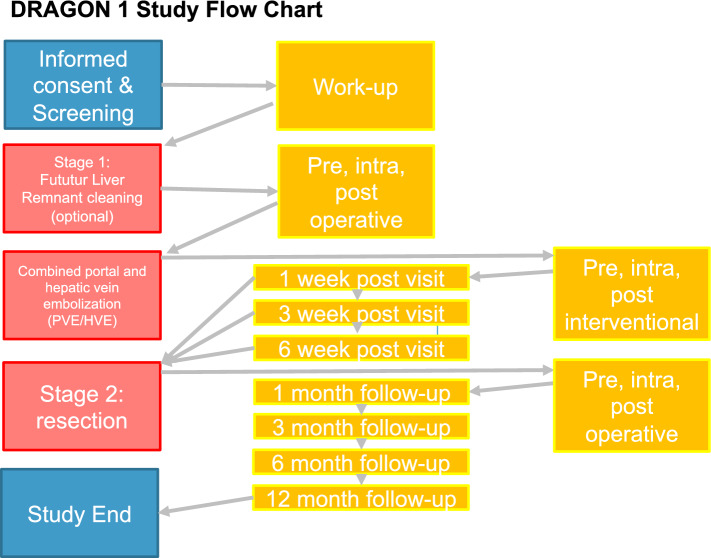
DRAGON 1 visit flowchart

After recruitment (*t* = 0), patient information and demographics are recorded. It is anticipated that a number of the included patients require a two-stage approach. The first step is to clean the FLR. A few days after, preferably in the same hospitalization, PVE/HVE is performed. One week after PVE/HVE, the first volumetry CT-scan is performed. If the FLR-volume is still insufficient, volumetry will be repeated at week three and week six. Once the FLR has reached a sufficient volume, resection is scheduled. After liver resection and the postoperative hospital stay, follow-up visits are scheduled at one, three, six, and twelve months. All diagnostic tests/ treatment procedures and visits are in accordance to standard clinical practice, except for the hepatic vein embolization during the intervention. All study visits are listed in the DRAGON 1 Flowchart (Fig. 1) and all study measures can be found in the SPIRIT Chart (Supplementary table 2).

### Primary Outcome

The primary outcome is a composite of two endpoints. Namely, the 90-day morbidity and mortality after PVE/HVE and the accrual of each participating center. Morbidity is assessed according to the Dindo-Clavien classification [22]. Accrual is defined as the time for each participating center from Site Initiation Visit (SIV) until 3 safe inclusions.

### Secondary Outcomes

Secondary endpoints comprise short- and long-term surgical and oncological outcomes. These include used neoadjuvant systemic treatment, PVE/HVE intra procedural data, FLR-hypertrophy, time to adequate FLR, resection rate, time to resection, intra operative data, number of oncological procedures performed besides PVE/HVE, recurrence, 1-year disease-free and overall survival.

### Sample Size

Prior to initiation, each participating center confirmed that a minimum of 3 inclusions within one year should be feasible. We expect that approximately 40 centers will be initiated in the DRAGON 1 trial. Therefore, the intended number of patients evaluated in the DRAGON 1 multicenter trial is *n* = 120 (40 centers times 3 patients per center). If the target of *n* = 120 is not reached, the trial will be evaluated regardless.

### Data Collection and Management

Pseudonymized data (coded by a study ID) will be entered in CASTOR secure online trials systems (Castor BV, Amsterdam, The Netherlands) and maintained by the Clinical Trials Center Maastricht. For further details see supplementary paragraph 4.

Data protection in the DRAGON 1 trial will be in compliance with the General Data Protection Regulation (EU).

### Statistical Analysis

For DRAGON 1, we will use descriptive coefficients to summarize outcomes. IBM SPSS Statistics will be used to display the results. A central interim analysis will be performed after enrollment of every 20 participants.

Access to the datasets used and analyzed during the study are available in a fully anonymized form from the sponsor upon reasonable request.

### Monitoring

Site Initiation Visit (SIV), Interim Visit, and Close Out Visit will be performed. As the DRAGON 1 trial has been categorized as medium risk, monitors will randomly check 25% of the data.

### Safety Assessment

All adverse and serious adverse events reported by the subject or observed by the investigator or staff will be recorded both in the Investigator Site File (ISF) and in CASTOR (supplementary paragraph 2). All complications will be categorized using the Dindo-Clavien classification.

A Data Safety Monitoring Board (DSMB) has been set up to guarantee independent evaluation of DRAGON 1 trial patients and to assist and advise Principal Investigators so as to protect the validity and credibility of the trial.

## Discussion

PVE/HVE is a new and promising percutaneous procedure to increase and accelerate the FLR-hypertrophy before resection with minimal physical impact for patients with primary (supplementary paragraph 5) and metastatic liver tumors.

Currently, new techniques are often implemented on single center level without appropriate scientific assessment. Consequently, data on safety or the indication of the new technique is often based on low quality observational studies and expert opinions. Technique development and safe implementation in consensus among expert centers is ideally required to prevent redundant studies or too liberal application.

The first prospective trial of the DRAGON trials collaboration, the single arm DRAGON 1 trial, aims to assess the safety profile of PVE/HVE in patients with CRLM and small FLR and the accrual potential of each participating center. It enables centers within the collaborative to gain experience based on consensus work instructions of PVE/HVE and consequently allow for safe implementation. Outcomes of the DRAGON 1 trial will be used to determine the effect size required for sample size calculation of the DRAGON 2 randomized controlled trial.

Furthermore, several technical approaches of PVE/HVE and different embolic agents used in PVE/HVE are described in literature. For the DRAGON 1 trial, it was decided in Delphi consensus (supplementary paragraph 3) to not standardize the Portal Vein Embolization procedure since these procedures are well established and, at time of writing the protocol, did not favor one approach over another. It was also decided not to use glue during HVE since glue migration was observed in cases within the collaborative group, albeit without clinical consequences.

To date, to avoid post hepatectomy liver failure, FLR function assessment seems to be more important than FLR volume to proceed with resection. Several modalities to measure total liver function are described, but currently Technetium-99 m (99mTc)-mebrofenin hepatobiliary scintigraphy (HBS) is the only reliable method to provide functional information of the FLR [23–25]. Interpretation of HBS is considered complex and time consuming, but more and more implemented in clinical pathways of major liver oncology centers. Unfortunately, at time of the start of DRAGON trial 1, multiple participating centers had not implemented HBS and only available data on liver function from participating sites performing HBS already is collected. Currently, all participating centers are encouraged to take part in the HBS implementation program, called “DRAGON meets HERCULES.” HBS data will be collected in a subset of centers during future DRAGON trials.

In the randomized DRAGON 2 trial, following the DRAGON 1 trial, in patients with CRLM and Primary liver tumors we will investigate the value of PVE/HVE over PVE alone in a superiority design. FLR hypertrophy, Kinetic Growth Rate, resectability, and survival among other outcomes will be studied.

### Trial Status

The growing DRAGON trials collaborative consists of more than 60 HPB centers.

The latest approved version of the DRAGON 1 trial protocol in Maastricht is version 4, April 21, 2021. The first informed consent was signed on May 8, 2020. Currently, 39 centers are actively recruiting patients in the DRAGON 1 trial. www.dragontrial.com can be consulted for the latest updates. The last patient will be recruited before July 1, 2022. The final report on the primary endpoint of the DRAGON 1 trial is expected by the end of 2022.

## Supplementary Information

Below is the link to the electronic supplementary material.

Supplementary file 1 (DOCX 170 kb)
