# Supplementary material for: Dragon 1 Protocol Manuscript: Training, Accreditation, Implementation and Safety Evaluation of Portal and Hepatic Vein Embolization (PVE/HVE) to Accelerate Future Liver Remnant (FLR) Hypertrophy
Source: Cardiovasc Intervent Radiol. 2022 Jul 5;45(9):1391–8. doi: 10.1007/s00270-022-03176-1 (PMC9458562; doi:10.1007/s00270-022-03176-1)
Supplement: Supplementary file 1 — (DOCX 170 kb) [file 270_2022_3176_MOESM1_ESM.docx]

**Supplementary Material**

**Supplementary paragraph 1: ALPPS background**
In 2012, ALPPS (Associating Liver Partition and Portal Vein Ligation for Staged Hepatectomy) was introduced, which showed a much higher resection rate of 92% compared to PVE (57%).^11-13^ However, high morbidity and mortality rates of 8% in the ALPPS group were also observed.

**Supplementary paragraph 2: AE/SAE reporting and recording**
All Adverse and Serious Adverse Events (AE/SAE) will be recorded by the centers. All events will be reviewed by the Data and Safety Monitoring Board (DSMB). There are no specific rules defined for early trial termination. Safety events include; Mortality after embolization or within 90 days after resection, complication after embolization or within 90 days after liver resection, pain after embolization and requirement of analgesia.

Adverse events are defined as any undesirable experience occurring to a subject during the study, whether related to the trial intervention or not. Additionally, all Serious Adverse Events will be reported to the METC azM/UM and if required to local ethical boards. Next to recording, SAEs will be reported to the accredited ethical research committee according to ERC guidelines. Additionally, both local PIs and coordinating PIs make a causality assessment of the event to the trial intervention based on the terms given in the ICH E2A guidelines (supplementary table 3). A work instruction has been created that includes all information on how to record and report (S)AEs events.

**Supplementary paragraph 3: Delphi consensus**
 in DRAGON Two Delphi rounds were initiated to homogenize interventions and the protocol. The protocol was subsequently written by the DRAGON management group. During an educational meeting it was decided only to accept staged procedures where PVE and HVE followed each other within 48 hours. Additionally, it was decided to only place Amplatzer Vascular Plugs (AVPs) in hepatic veins without using glue, a method we call Portal and Hepatic Vein embolization (PVE/HVE).^21^ Given the paucity of published data at time of protocol writing, it remains unclear, however, which method is best to induce maximal liver regeneration with minimal risk.

A training procedure was developed by the study team, which must be followed by all of the designated study personnel at each center before the center can be initiated. Work Instructions (WIs) were created in order to improve adherence to the intervention and DRAGON 1 study tasks (e.g. Data entering, DRAGON image bank, etc.). These WIs were sent to each center.

**Supplementary paragraph 4: Data collection detailed**Identifying information such as names, dates of birth, addresses and e-mail addresses or web addresses will not be entered. The internet-based secure data management trials system CASTOR will also be used for data and query management, monitoring, reporting and coding. All patient’s imaging during the study period will be recorded in the DRAGON trial 1 image bank. The de-identified scans will be uploaded in a password protected and secured online storage drive. Essential documents must be retained according to local law for at least 15 years after the regular end or a premature termination of the respective study.

**Supplementary paragraph 5: PVE/HVE in patients with primary tumors**
Due to the worldwide lack of experience with PVE/HVE in primary liver tumors and the fear of tumor necrosis, due to higher vascular dependency of tumors in often a diseased liver, primary liver tumors are not included in the DRAGON 1 trial. This was a consensus decision after multiple Delphi rounds among 40 surgical and radiological experts within the DRAGON collaborative. Currently, growing experience and insights within the DRAGON collaborative suggests that PVE/HVE can be safely performed in patients with primary liver tumors as well. Future DRAGON trials will be initiated for patients with primary liver tumors.

**Handling Missing data
I**n case of missing data, several methods will be considered to handle missing data depending on which variables are missing, the mechanism of incomplete data (i.e. Missing Completely At Random (MCAR), Missing At Random (MAR), and Missing Not At Random (MNAR)), and the missing rate. Complete Case Analyses can be considered if the percentage of missing values is low (e.g. <10%). Mean imputation can be used for covariate missingness. For outcome missingness exceeding 10%, multiple imputation can be used.

**Supplementary Tables**

| **Country** | **Center** |
| --- | --- |
| **Germany** | Klinikum Saarbrücken |
|  | University Hospital Aachen |
|  | University Hospital Halle (Saale) |
|  | University Hospital Frankfurt |
|  | University Hospital Bonn |
|  | University Hospital Heidelberg |
| **Italy** | Ospedale San Raffaele, Milan |
|  | Fondazione Poliambulanza Brescia |
|  | Gemelli university hospital Rome |
|  | Maggiore Hospital, Bologna |
|  | Sant'Orsola-malpighi hospital, Bologna |
| **Spain** | University Hospital Germans Trias I pujol, Badalona |
|  | Hospital Universitari Dr. Josep Trueta de Girona |
|  | Hospital Taulí, sabadell |
|  | Hospital Universitari Mútua Terassa |
|  | University hospital Miguel Servet, Zarragoza |
|  | Clinic, Barcelona |
| **Belgium** | CHU liège |
|  | Hôpital Erasme, Brussels |
|  | CHU-UCL Namur site Godinne (UCLouvain) |
| **UK** | Queen Elizabeth Hospital, Birmingham |
|  | Royal Free Hospital, London |
|  | King’s college Hospital, London |
|  | University hospital Southampton |
|  | the Royal Bournemouth and Christchurch Hospitals |
|  | Aintree University Hospital |
|  | Newcastle-upon-tyne Hospitals |
|  | Oxford University Hospitals |
| **USA** | Memorial Sloan Kettering Cancer Center, New York |
|  | Rush University Medical Center, Chicago |
|  | Yale school of medicine hospital, New Haven |
| **Canada** | L'hopital d'Ottawa |
|  | McGill University Health Centre, Montreal |
| **Australia** | Monash Health, Clayton |
|  | Westen Health, Footscray |
|  | Royal prince Alfred Hospital, Sydney |
|  | Royal Brisbane Hospital |
| **The Netherlands** | Maastricht University Medical Center |
|  | University Medical Center Groningen |
|  | University Medical Center Utrecht |
|  | Amsterdam University Medical Centers, Location AMC |
|  | Amsterdam University Medical Centers, Location VU |
|  | Erasmus Medical Center, Rotterdam |
|  | Maxima Medisch Centrum, Eindhoven |
|  | Amphia, Breda |
| **Norway** | University Hospital Oslo |
| **Switzerland** | KSW Winterthur |
|  | St. Claraspital and Clarunis University Hospital Basel |
| **Sweden** | University Hospital Linköping |
|  | Karolinska University Hospital Stockholm |
| **China** | Zhongshan Hospital, Fundan university, Shanghai |
| **Austria** | Social Center South, Vienna |

**Supplementary table 1:** Overview of participating centers


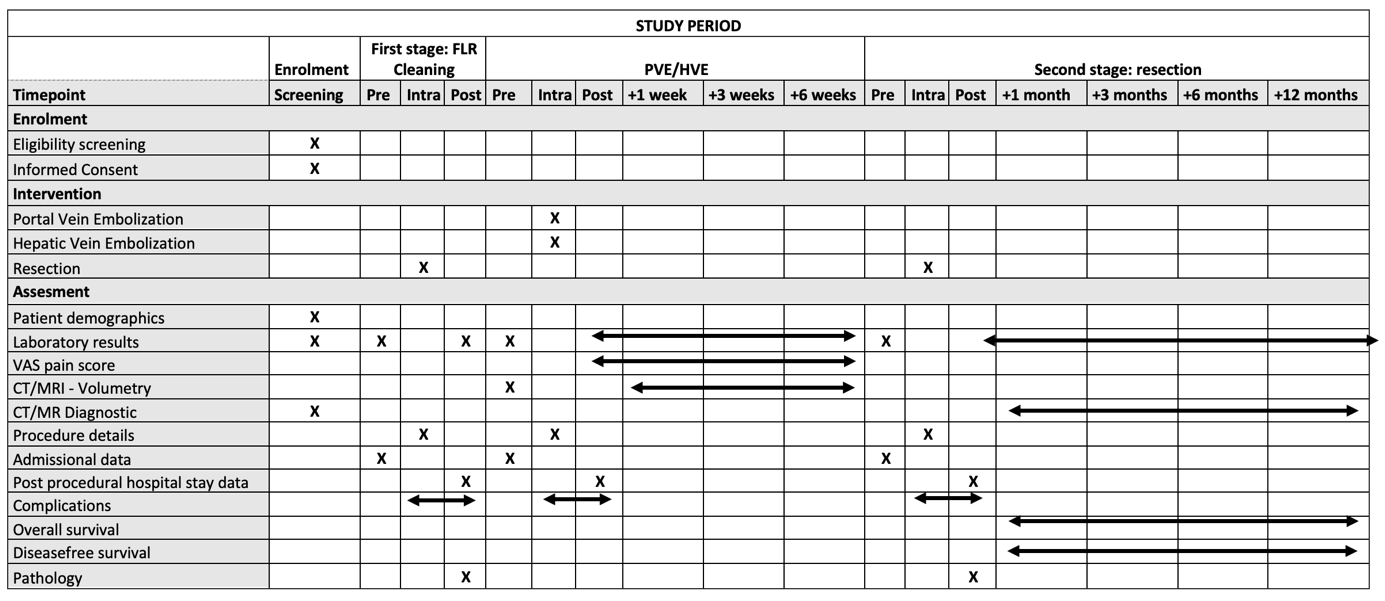
**Supplementary table 2:** SPIRIT chart

| Relationship | Description |
| --- | --- |
| Definitely | Temporal relationship |
| Probably | Temporal relationship  No other cause evident |
| Possibly | Temporal relationship  Other cause possible |
| Unlikely | Any assessable reaction that does not fulfil the above conditions |
| Not related | Causal relationship can be ruled out |

**Supplementary table 3:** causality assessment
